# *Vis à Vis*. Mentoring processes and responsibility in juvenile justice and residential youth care centers: preliminary results

**DOI:** 10.3389/fpsyg.2026.1850698

**Published:** 2026-07-08

**Authors:** Valeria Saladino, Angela Rosa Marchio, Chiara Eva Blandi, Anna Cappa

**Affiliations:** 1 “Magna Graecia” University of Catanzaro, Catanzaro, Italy; 2 Psicotypo - Mental Health Professionals ODV, Rieti, Italy

**Keywords:** deviance, justice-involved juveniles, mentoring, recidivism, social rehabilitation

## Abstract

Justice-involved juveniles frequently present significant social and psychological difficulties linked to complex trauma, insecure attachment, and adverse childhood experiences (ACEs), which may impair emotional regulation, identity development, and relational functioning, increasing the risk of antisocial behavior. Within this framework, the *Vis à Vis* project—developed under the European Researchers' Night initiative *SuperScienceMe*—aims to promote future-oriented planning and personal change among at-risk youth (14–25) through integrated psychological and educational support. Its objectives include enhancing empowerment and self-regulation, fostering responsibility and awareness of behavioral consequences, and promoting restorative justice through reparative actions and social inclusion. Implemented by the “Magna Graecia” University of Catanzaro and authorized by the Italian Ministry of Justice, the project involved 12 participants (5 juveniles and 7 student mentors) in structured experiential activities under professional supervision, following a multi-phase design. Preliminary findings indicate increased self-awareness, improved interpersonal skills, greater accountability, and enhanced future orientation among juveniles involved. Despite its exploratory nature, the project highlights the value of mentoring-based, restorative approaches in addressing developmental vulnerabilities and supporting positive behavioral change.

## 1 Introduction

Justice-involved juvenile often present social and psychological characteristics marked by reduced emotional regulation capacities, difficulties in identity formation, and deficits in relational and mentalization processes (Fonagy et al., 2002). These conditions are frequently associated with traumatic experiences, insecure attachment patterns, and childhood neglect (Kerig and Becker, 2010; Jewell et al., 2015). Specifically, regarding complex trauma, the literature highlights that prolonged and cumulative exposure to relationally traumatic experiences—such as neglect, abuse, family instability, and violence—pervasively impacts affect regulation mechanisms, relational skills, and identity organization. Unlike single traumatic events, complex trauma is characterized by repeated experiences, most often stemming from the primary attachment relationship, with dysfunctional and disorganized effects on individual development (Cook et al., 2005; van der Kolk, 2005).

Consistent with this perspective, more recent studies on Adverse Childhood Experiences (ACEs) confirm that cumulative exposure to adverse events during development is strongly associated with psychopathological outcomes, emotional dysregulation, risk behaviors, and increased involvement in antisocial conduct during adolescence and adulthood (Felitti et al., 1998; Hughes et al., 2017; Graf et al., 2021; Malvaso et al., 2022). The impact of such exposure can be conceptualized as dose-dependent: the greater the exposure to traumatic events, the more severe the pathological consequences. This suggests that the accumulation of traumatic factors progressively impairs self-regulatory and adaptive capacities (Kaysen et al., 2010). This theoretical framework is supported by recent neurobiological evidence showing that chronic early stress and traumatic exposure are associated with dysregulation of the hypothalamic–pituitary–adrenal (HPA) axis and long-term structural and functional alterations in brain regions involved in emotional regulation, impulse control, and social cognition, including the amygdala, hippocampus, and prefrontal cortex (Teicher and Samson, 2016; McLaughlin et al., 2020; Gee, 2016; McCrory et al., 2010).

Consequently, deviant behavior, rather than being understood as a deliberate transgressive choice, can be interpreted as a reflection of impairments in mentalization and affect regulation processes (Fonagy and Luyten, 2009). Mentalization—the capacity to understand and interpret one's own and others' behavior in terms of intentional mental states—plays a crucial role in the modulation of social conduct and impulse control. Mentalization is not a stable, static process; rather, it is dynamic, varying according to emotional arousal and relational context. For this reason, in situations of interpersonal stress, particularly in individuals with insecure or traumatic attachment, mentalization capacity may collapse, giving rise to pre-mentalizing modes of functioning (Fonagy and Target, 2001; Bateman and Fonagy, 2016).

These modes include “hypomentalizing,” characterized by a reduced capacity to associate complex mental states with oneself and others through simplified, superficial interpretations of behavior, and “hypermentalizing,” which involves over-attributing and distorting one's own and others' mental states, sometimes incorporating elements of suspicious or persecutory interpretations (Sharp et al., 2011b).

According to recent literature, both hypomentalizing and hypermentalizing modes are associated with reactive aggression, emotional dysregulation, and antisocial behaviors during adolescence, particularly in youths exposed to traumatic relational environments (Sharp et al., 2011a; Ensink et al., 2017; Luyten et al., 2020).

Specifically, deficits in mentalization- ability to understand and interpret one's own and others' mental states in terms of emotions, thoughts, and intentions- under conditions of excessive affective arousal can increase hostile attribution bias, leading to more impulsive, action-oriented responses rather than reflective ones (Fonagy and Bateman, 2016; Choi-Kain and Gunderson, 2008). From this perspective, deviant behavior can be considered not as a consequence of moral deficiency, but as the result of disruptions in reflective processes that mediate experience, emotional state, and behavior (Dodge et al., 1990; Taubner et al., 2013).

Adolescence represents a particularly sensitive period for the development of mentalization capacities, coinciding with a reorganization of attachment systems and increasingly complex relational demands. Pre-existing vulnerabilities in mentalization processes may be amplified during this developmental stage, particularly when combined with social exclusion, marginalization, and exposure to deviant models (Fonagy and Luyten, 2009).

In this context, juvenile justice models oriented toward rehabilitation and re-education are particularly effective in promoting identity development, emotional competence, and social skills, facilitating social reintegration, and contributing to recidivism reduction (Mulvey et al., 2010; Lipsey, 2009). Criminological studies indicate that educational and rehabilitative approaches, including family-based treatment programs and restorative justice initiatives, are associated with significant reductions in recidivism among juveniles compared to groups without structured interventions (Schwalbe et al., 2012; Lipsey and Cullen, 2007). Conversely, youths processed or treated in more punitive contexts tend to exhibit higher recidivism rates (Myers, 2003).

Within this framework, mentoring emerges as both an individualized accompaniment technique and a relationally transformative tool. Mentoring can influence identity development and emotion regulation in justice-involved juveniles. Rhodes (2005) identifies three primary areas of developmental impact: socio-emotional, identity-related, and cognitive. Theoretically, mentoring can be interpreted through attachment theory and socio-relational developmental models. In cases of insecure or disorganized attachment, a new relationship with a significant adult outside the family context can serve as a corrective experience, functioning as a substitute secure base (Rhodes, 2002).

The mentor, however, should not replace parental figures but can offer a stable relational space in which the youth can experience trust and affect regulation.

Empirical evidence supports the perspective that this process is particularly relevant for individuals with complex trauma. A meta-analysis by (DuBois et al. 2011), focusing on mentoring programs for youth at risk of delinquency, found significant effects on behavioral, academic, and relational outcomes.

Structured mentoring programs—characterized by mentor training, relational continuity, clear objectives, and clinical supervision—have been associated with improved emotion regulation, reductions in antisocial behaviors, and stronger social competencies among justice-involved youth DuBois et al., (2011; Raposa et al., 2019; Cavell et al., 2021).

These findings suggest that mere adult presence is insufficient to produce change. Indeed, the quality, duration, and intentionality of the educational relationship are critical.

Within juvenile justice contexts, mentoring aligns coherently with restorative and re-educational models. Additionally, structured individual mentoring programs have been shown to correlate with reduced recidivism, particularly when combined with family and community interventions (Lipsey, 2009; Schwalbe et al., 2012).

Mentoring influences representational processes, contributing to the restructuring of internal working models and the construction of a self-narrative less centered on delinquency and stigmatization (Rhodes, 2015). It does not merely provide pragmatic support but fosters a potentially transformative relational experience capable of impacting the psychological processes underlying deviant behavior (Maruna, 2001).

Additionally, group interventions are a fundamental tool in preventive and rehabilitative processes for justice-involved juveniles, particularly when integrated into structured programs aligned with theoretical principles such as mentalization, emotion regulation, and attachment. Group interventions promote peer comparison, social skills development, and modification of dysfunctional behavioral patterns (Ferrara, 1992; Jewell et al., 2015). Group approaches allow participants to work on relational dynamics in a mutual-support context, where observation and interaction with others foster prosocial skills development (Sequeira Nazaré and Schmitz, 2025).

Recent studies suggest that structured group interventions grounded in restorative and trauma-informed approaches can promote emotion regulation, peer support, prosocial identity development, and reductions in aggressive and antisocial behaviors among justice-involved adolescents (Jewell et al., 2015; Sequeira Nazaré and Schmitz, 2025; Olaghere et al., 2021).

The literature highlights that some group models can positively influence recidivism rates among justice-involved juveniles. Specifically, a longitudinal study showed that group participants were less likely to reoffend compared to control groups (Jewell et al., 2015). For group interventions to be effective, however, proper training of facilitators and clinical supervision is essential. Additionally, careful management of the setting is critical, ensuring a safe environment that guides relational processes constructively.

The *Vis à Vis* project is specifically based on this approach, addressing the “interrupted” developmental pathways of youth within the correctional setting, with the goal of transforming self- and other perception through direct human interaction. Its innovation lies in bridging the gap between academic research and the community, delivering specialized psychological support directly within juvenile facilities to allow youth to observe their life narratives through the mirror of a supportive relationship.

Simultaneously, the integration of group interventions maximizes the relational experience, fostering social learning, prosocial skill development, and shared experiences in safe contexts where youth can engage in cooperative dynamics and interact with peers facing similar challenges.

A further distinctive feature of the project is the involvement of students and graduate trainees in forensic and criminological psychology, who refine their comprehension and reflective skills beyond the simple role of mentor. These students apply theoretical knowledge acquired through their academic studies in practical observation and active involvement with justice-involved juveniles, constructing individual mentoring relationships and group dynamics. The aim is to create a “dyadic” relationship within the group, maintaining each participant's individuality while situating them in a collective context of growth and exchange.

In this framework, the project does not merely offer generic mentoring but develops a psychologically and academically informed intervention capable of producing tangible changes in the emotional, relational, and behavioral processes of youth, valuing both the individual and collective dimensions.

The theoretical frameworks discussed above are integrated into a coherent conceptual model guiding the intervention design. Accordingly, the *Vis à Vis* project operationalizes attachment-based, mentalization-informed, and restorative justice principles within a structured relational and group-based setting.

Indeed, the project is explicitly grounded in restorative justice principles, which are systematically operationalized throughout its core components. In particular, accountability is fostered through structured reflective exercises that invite participants to acknowledge their actions and their impact on others within a guided and non-judgmental relational setting. Harm awareness is developed through group-based discussions and narrative activities that encourage youths to critically reflect on personal experiences of damage, vulnerability, and relational rupture. Relational repair is promoted through the mentoring dyad and group interactions, which function as contained relational spaces in which trust, recognition, and corrective emotional experiences can emerge. Finally, social reintegration is supported by activities aimed at reconstructing personal narratives and strengthening prosocial identity, allowing participants to reframe their self-perception beyond stigmatizing labels. Through this multi-layered structure, restorative justice is not treated as a theoretical framework, but as an applied methodological orientation embedded in mentoring practices, group work, and reflective exercises that collectively aim to transform relational patterns and support psychosocial reintegration.

## 2 Objectives

The *Vis à Vis* project aims to foster social inclusion for minors and young adults (ages 14–25) who are detained in juvenile justice institutions, residing in residential communities, or engaged with youth-at-risk services. Specifically, it seeks to encourage the development of personal projects grounded in awareness of illicit behaviors and their consequences, alongside a motivational pathway oriented toward individual change and the cessation of risky or deviant behaviors.

More specifically, the project pursues the following objectives:

- Empowerment: Facilitate individualized pathways of personal change toward crime abstention by strengthening resilience mechanisms and emotional regulation in relation to behavioral choices. This process encourages participants to move from initial distrust to active engagement, motivating them to rediscover and invest in their personal resources to build a positive social identity for their future beyond institutional or community contexts.
- Responsibility: Promote participants' awareness of the impact of criminal behavior on victims and the broader community, encouraging them to take responsibility for past actions and future choices. By providing a reflective space, the project helps youth shift from guilt to constructive “responsibility,” allowing them to visualize the consequences of their actions on others.
- Restorative Action: Motivate and support participants in actively addressing the consequences of their offenses by developing and implementing reparative behaviors (including symbolic forms of restitution) toward victims and the community. In this sense, the project functions as a cultural advocacy initiative, countering the stigmatization of young offenders and promoting a culture of legality and restorative justice.

## 3 Materials and methods

### 3.1 Procedures

The *Vis à Vis* project was sponsored as part of the “SuperScienceMe—Research is your Elevation,” a European Researchers' Night initiative involving the University of Calabria, “Magna Graecia” University, Mediterranean University, University of Basilicata, the National Research Council (CNR), and the Calabria Region. It is one of nine Italian projects funded by the European Commission for the 2024–2025 period and aims to redefine the relationship between art, science, and society, with a strong focus on sustainability, inclusion, and community engagement.

Beyond these general objectives, the projects presented within “SuperScienceMe” are specifically intended to promote social inclusion and strengthen dialogue between academic institutions and vulnerable communities. Within this framework, the research team from the “Magna Graecia” University of Catanzaro developed a community-oriented intervention involving justice-involved juveniles hosted in a ministerial residential community. The project was authorized by the Italian Ministry of Justice. Throughout all project phases, the juveniles were accompanied and supervised by the relevant legal and community professionals, who ensured voluntary participation, ethical protection, and participant safety. The *Vis à Vis* project was also the subject of a dedicated competition and received first prize for originality and social impact.

Consistent with these premises, the initiative was conceived primarily as an inclusion and engagement project aimed at fostering a communicative and relational bridge between the university environment and juveniles living in conditions of psychosocial vulnerability and marginalization. Accordingly, the intervention was designed from a community-based and participatory perspective rather than as a traditional investigator-driven experimental study focused on the statistical validation of predefined outcomes.

For this reason, the methodological design did not include standardized psychometric instruments or rigid quantitative outcome measures. The decision to avoid formal statistical assessment procedures was intentional and reflected both the exploratory nature of the initiative and the need to preserve a flexible, relational, and non-clinical setting capable of encouraging trust, participation, and spontaneous interaction among participants. Within community-oriented interventions involving vulnerable populations, excessively structured evaluative procedures may risk interfering with the development of authentic relational dynamics and with the inclusive objectives of the project itself.

Instead, the intervention was informed through qualitative and descriptive sources of information. Specifically, the authors examined the participants' legal and social case files and conducted interviews with community professionals and operators working directly with the juveniles. These procedures allowed the collection of contextual and descriptive data regarding participants' psychosocial backgrounds and facilitated the identification of common relational and communicative needs across the group. Based on this preliminary assessment, the intervention primarily focused on two core dimensions shared by participants: relationship with others and communication.

Therefore, the findings reported in the present manuscript should be interpreted as preliminary, descriptive, and practice-oriented outcomes that document the feasibility, acceptability, and social relevance of the intervention, rather than as statistically validated evidence of efficacy. This choice was consistent with the aim of capturing relational, behavioral, and experiential changes emerging during the intervention, rather than measuring clinical outcomes through standardized psychometric instruments. Accordingly, effectiveness was interpreted in terms of observable qualitative indicators such as engagement, emotional expression, reflective capacity, and interpersonal interaction.

In addition, the feedback sessions and debriefings conducted with mentors, community professionals, and the juveniles themselves were not intended to serve a statistical or psychometric purpose, but rather an operational and formative one, aimed at continuously adapting and implementing the intervention according to participants' emerging relational and communicative needs. Consequently, the findings reported in the present manuscript should be interpreted as preliminary, descriptive, and practice-oriented outcomes documenting the feasibility, acceptability, and social relevance of the intervention, rather than as statistically validated evidence of efficacy. Nevertheless, this methodological approach does not exclude the possibility that future implementations of the project may incorporate more structured and rigorous assessment tools and quantitative outcome measures in order to further evaluate and strengthen the effectiveness of the intervention.

### 3.2 Participants

The project involves both qualified professionals and students enrolled in the master's program in Forensic and Criminological Psychology at the “Magna Graecia” University of Catanzaro.

Mentors participate in structured cycle of meetings aimed at enhancing transversal, relational, and social competencies of both students and the justice-involved juveniles.

The project involved a total of 12 participants—five male justice-involved juveniles and seven mentors (85% females) The juveniles hosted in the ministerial residential community were predominantly male and presented personal histories characterized by significant psychological and social vulnerabilities. Through the analysis of legal and socio-educational case files, integrated with interviews and discussions with community professionals, a shared background of traumatic experiences and dysfunctional family relationships emerged. Specifically, juvenile participants reported histories of neglect, family instability, exposure to violence, experiences of abandonment, and early relational difficulties, elements consistent with theoretical models of complex trauma and Adverse Childhood Experiences (ACEs). Although no standardized trauma assessment was administered, the analysis of legal and socio-educational case files, together with interviews with community professionals, suggested that all participating juveniles had experienced at least one form of significant relational or environmental adversity consistent with trauma-related backgrounds and Adverse Childhood Experiences (ACEs).

The exclusion of youths with psychiatric diagnoses does not reflect a denial of the high prevalence of mental disorders within juvenile justice and residential care settings, which is widely documented in the international literature (Teplin et al., 2002; Fazel et al., 2008). Rather, this decision was primarily related to the exploratory, relational, and non-clinical nature of the intervention. Since the project was not designed as a specialized therapeutic setting and did not include structured clinical procedures for the management of severe psychopathological conditions, only participants able to engage in the experiential and group-based activities without the need for intensive psychiatric support were included. This methodological choice was intended to ensure participants' psychological safety, preserve the relational balance of the group, and maintain consistency with the educational and community-based aims of the project. The project was conducted under the direct supervision and guidance of the Principal Investigator (PI) who actively monitored and managed all sessions while leading the group.

No formal fidelity check procedures or standardized implementation adherence measures were adopted. However, intervention delivery was continuously monitored through direct supervision by the Principal Investigator and structured debriefing sessions with mentors and community professionals, ensuring consistency with the planned protocol.

### 3.3 Project phases

This study adopts a preliminary qualitative and community-based exploratory design. To ensure organizational and methodological clarity, the project follows these phases:

Phase 0: Information meeting with the institutions involved and coordination with teaching staff. The project is part of a community context with a ministerial setting. The aim of this phase is to illustrate the project's objectives to the team, the facility's staff, and the teaching staff to ensure its feasibility and institutional alignment.

Phase 1: Identification and selection of participants by institutional administrators and faculty, following the team's presentation of the project, based on predefined inclusion criteria: understanding of the Italian language and absence of a psychiatric diagnosis. Data collection took place through the analysis of participants' legal and social files and the conduct of fact-finding interviews with community professionals and workers. At the end of this phase the team plan the activities.

Phase 2: Conducting experiential couple and group activities over the course of 3 days. The working sessions were structured according to a defined framework aimed at promoting collaboration and the psychological wellbeing of the participants. Each meeting began with a welcoming moment and an emotional check-in: after the students had taken their seats and the mentor had introduced the activity, a dedicated space was provided to explore the students' emotional states. This initial phase helped foster a climate of mutual listening and validation, preparing the group for the activity. Subsequently, the mentor presented the day's activity, defining the relational setting between the team and the participants. Depending on the objectives, team members positioned themselves either in dyads (face-to-face with individual participants) or in a group arrangement, actively engaging in the activity alongside the participants. This direct involvement of the team served both modeling and scaffolding functions, with the aim of supporting participants in areas of greater vulnerability and helping them address the specific difficulties that emerged during the activities.

The final phase of each exercise was characterized by a moment of phenomenological sharing and debriefing. Each participant, including the team members, was invited to present and/or read their work aloud, encouraging the social sharing of the produced content. Immediately afterward, the discussion shifted toward affective processing, exploring the emotions elicited by the activity. In this context, the team's self-disclosure played a crucial role in creating a reassuring and non-hierarchical environment, facilitating emotional expression even among participants who initially showed resistance or communication barriers.

Each meeting concluded with a final synthesis and structured feedback session aimed at gathering the children's impressions and summarizing the meanings that emerged, thereby consolidating the experience lived throughout the day. At this stage, a predominantly observational approach was adopted. During dyadic or group activities, participants' relational dynamics, behaviors, and interaction patterns were monitored in real time.

Phase 3: Structured qualitative feedback was collected from both participants and the university students involved in the project. A qualitative approach was employed during the sharing sessions held at the end of the activities to explore participants' subjective experiences and understand the emotional and experiential impact of the intervention, including its perceived usefulness and relevance. An observational and relationship-based qualitative approach was adopted because the intervention was not conceived as a mere administration of tasks, but rather as a relational and clinically oriented support process focused on listening to emotional experiences and strengthening individual resources through sharing and reflection within the group and with the team.

Phase 4: Follow-up based on collected qualitative feedback, through reflective discussions with participants, students, and institutional staff to identify strengths, areas for improvement, and potential directions for future implementation.

Phase 5: Project presentation and competition.

### 3.4 Activities

All phases of the project were supervised and monitored by professionals working within the ministerial residential community, ensuring continuous debriefing sessions and feedback both before and after each meeting.

The project includes 3 days (2 h long) of experiential and reflective group activities conducted over 3 weeks, following a relational progression from the concept of individual “I” to the relation with another person “You” and finally to the community “We.”

In this frame, activities are designed to foster interpersonal connection, self-awareness, emotional expression, and future-oriented reflection through guided individual, dyadic, and group formats.

Participants are selected according to the following inclusion criteria: (a) absence of diagnosed psychiatric disorders requiring specialized clinical treatment, and (b) adequate comprehension of Italian to ensure meaningful participation in relational and reflective activities.

Activities are conducted using a mixed format (dyadic and group) as follows:

**Getting to Know Each Other and Building Friendships:** Participants create a personal “identity card,” including selected information about themselves. Specifically, the card requires participants to disclose their name, date and place of birth, passions, and personal strengths and weakness. Furthermore, it encourages deeper self-reflection by asking for a description of a fun life experience, personal fears and future dreams. This group activity is followed by guided sharing and dialogue. Using drawing as a primary expressive tool, youth share personal fragments in a supportive context, facilitating mutual understanding, dialogue, and self-disclosure.

**Representing Ourselves Through Our Name:** Working in pairs, participants write their names on a white paper. Each letter is associated with a word or adjective that represents them. Partecipants are encouraged asking each other for help and suggestions, allowing them to reflect on their own identity through the eyes of the other. Each pair then cuts out a fragment of the partner's sheet, selecting an adjective to keep as a symbolic reminder. The activity encourages reflection on personal values and qualities and fosters recognition of what is meaningful for oneself and others by transforming self-perception into a shared relational experience.

**Putting Yourself in Someone Else's Shoes:** Participants sit facing each other, hold hands, and engage in synchronized breathing. This exercise emphasizes bodily “vibrations” and non-verbal attunement. Afterwards, each participant traces their hand on a white paper and writes within the partner's hand outline what they perceived and received during the shared experience. The activity fosters openness, self-awareness, and expression of thoughts, emotions, and bodily sensations through non-verbal interaction, helping overcome physical barriers and providing deep emotional containment.

**Offering a Part of Myself:** Participants write one letter to their past self and another to their future self, promoting reconciliation with their personal history. From these texts, participants select significant excerpts to share and discuss collectively. Individual fragments were used to create an audio-recorded narrative shared within the members of the group. The activity encourages self-awareness and supports the construction of a coherent, future-oriented self-narrative, fostering reflection on identity, change, and personal goals (Table 1).

**Table 1:** Resume of the main activities, objectives, and results.

Activity	Objectives	Outcome	Criticality
First session “I”			
15.6-7.4,-51.5499pt Getting to know each other and building friendships	Facilitate mutual understanding, foster interpersonal dialogue, encourage self-disclosure, provide a place for narrative sharing of personal experiences, and mitigate initial social barriers.	All sections were completed with mentor support. The drawing tool successfully enabled reserved participants to communicate additional personal descriptive elements beyond verbal language.	Initial social inhibition and embarrassment. The transition from a formal institutional climate to a shared relational space required significant emotional mediation.
Second session “You”			
15.6-7.4,-51.5499pt Representing ourselves through our name	Assign value to aspects of one's identity that are personally significant, while fostering the recognition and validation of these core attributes within a relational exchange.	The symbolic exchange established a more trusting and relaxed atmosphere. Participants felt recognized, strengthening the relational bond with mentors.	Significant difficulty in identifying personal attributes, both positive and negative. Low self-esteem and self-judgment made deep introspection challenging without mentor guidance.
Third session “We”			
Putting yourself in someone else's shoes I give you a part of myself	Promote interpersonal openness, enhance self-awareness, and facilitate the reciprocal transmission of thoughts, feelings, and bodily sensations through non-verbal contact. Cultivate awareness of one's past history in order to define the person they aspire to become in the future.	A strong emotional attunement was achieved. Participants successfully transmitted both positive and negative sensations to their partners based on their current moods, facilitating a deep and authentic interpersonal connection. Participants experienced a strong sense of commonality and shared identity. The collective sharing of personal letters fostered a supportive environment where youth felt less isolated and more motivated toward personal change.	High levels of initial physical embarrassment and discomfort. The direct contact and the vulnerability of the exercise caused significant unease for some participants, requiring careful emotional containment by the mentors. Confronting past traumas and future uncertainties was emotionally demanding. Some participants experienced significant difficulty in processing painful memories, requiring continuous emotional support to maintain their focus on a constructive future orientation.

## 4 Results

**Figure 1:**
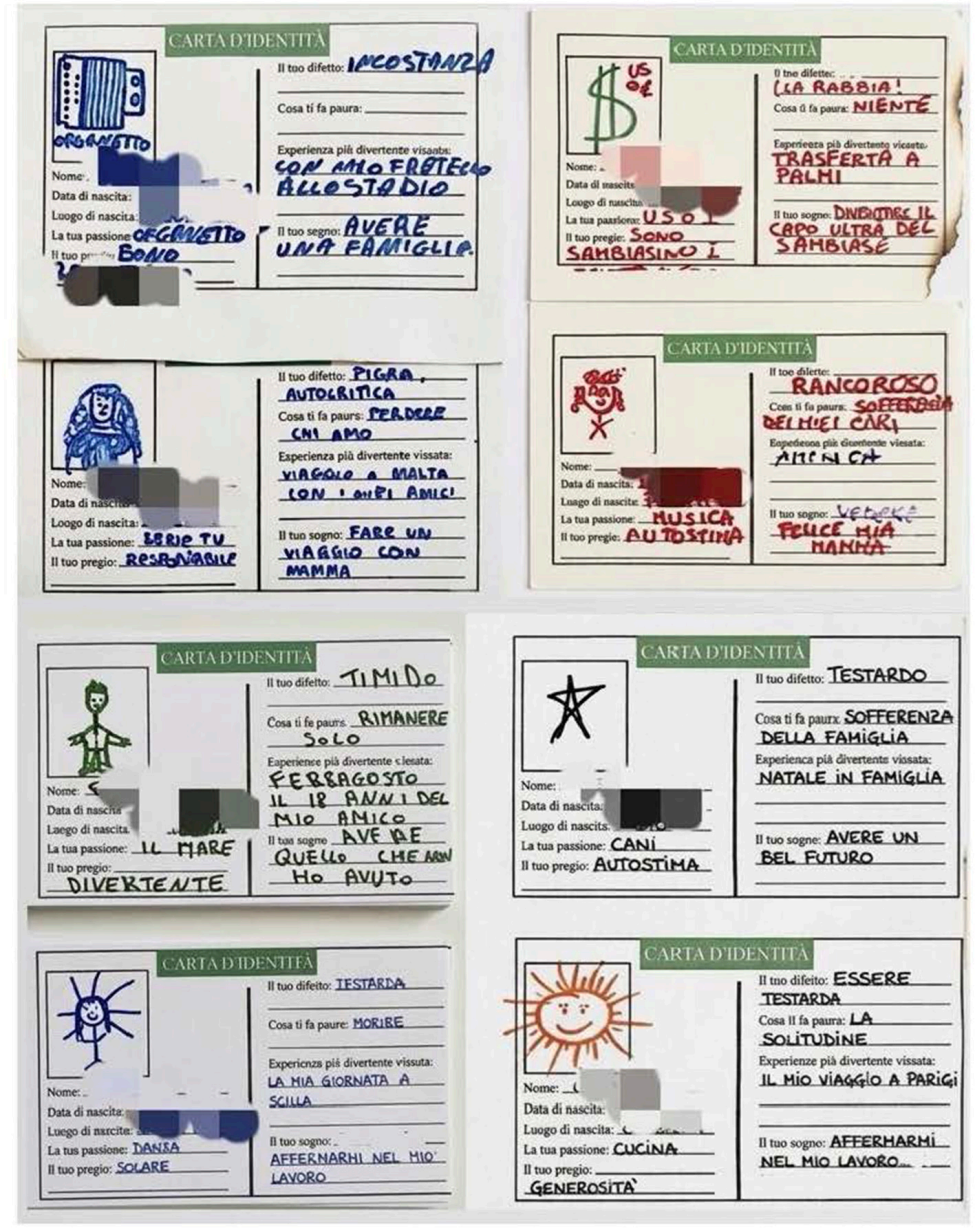
Getting to know each other and building friendships.

The results show how the different activities concretely contributed to achieving the project's specific objectives. In particular, the first activity (“Getting to Know Each Other and Building Friendships”), through the completion of the Identity Card, represented a fundamental first step toward empowerment, fostering the emergence of identity-related elements and initiating a process of self-reflection. Despite initial difficulties related to embarrassment and social inhibition, participants gradually began to recognize and share personal aspects, laying the groundwork for greater active engagement. Observations indicated that participants initially adopted a defensive stance, using humor and irony to downplay the significance of the activity. The presence of mentors from the Forensic and Criminological Psychology Master's program proved pivotal; as one mentor noted, “Seeing us become personally involved encouraged them to lower their guard.” A significant moment occurred during the “fears” section, when one participant privately shared with his mentor: “I wanted to write more, but these topics are too delicate to face right now.” This suggests that, while the creative drawing activity helped break the ice, some emotional themes required a greater level of established trust before participants felt able to disclose them fully (Figure 1).

**Figure 2:**
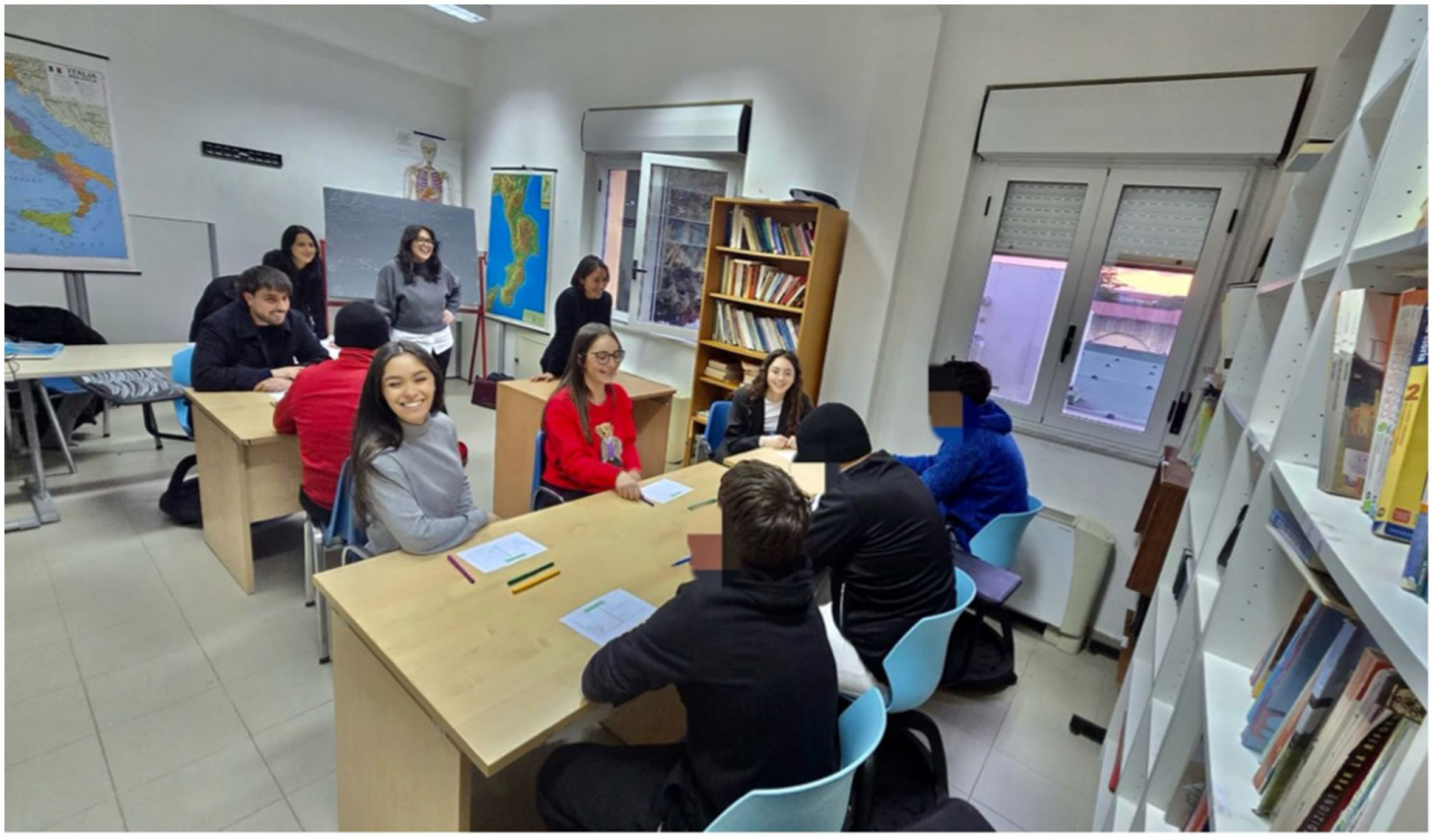
Representing ourselves through our name.

The second activity (“Representing oneself through one's name”) further strengthened the empowerment process and introduced a meaningful dimension related to responsibility, understood as self-awareness and recognition of one's own value within relationships with others. At this stage, the focus shifted to mutual recognition within the relational dyad, with the aim of highlighting the most significant identity aspects and, at the same time, fostering their validation through interaction with others. From an operational perspective, participants were asked to create an acrostic using the letters of their name, associating each letter with a distinctive personal trait, characteristic, or concept expressed through an adjective or noun. This symbolic exchange proved to be highly effective, contributing to the creation of a more trusting, empathetic, and relaxed group atmosphere. Participants reported feeling genuinely recognized by others, which also significantly strengthened their relational bond with the mentors. However, a critical issue emerged regarding self-perception: many participants experienced considerable difficulty in identifying their own personal characteristics, both positive and negative. This widespread fragility in self-esteem, along with a tendency toward harsh self-judgment, made the process of introspection challenging, highlighting how activities of this kind require strong guided support and continuous facilitation by mentors to be truly effective. A significant emotional blockage emerged during the individual task. Several participants remained staring at their names, unable to identify even a single adjective to describe themselves. To mask their discomfort and embarrassment, they resorted to loud laughter and disruptive joking. As one mentor observed, “The difficulty was not simply finding a word, but accepting that a positive word could belong to them.” The mentors intervened by suggesting traits they had observed during the previous hours. The atmosphere shifted dramatically during the symbolic exchange: after reading the adjective chosen for him by his mentor, one participant looked up in surprise and whispered, “You're right… I had never thought about myself this way before.” This moment suggests that external validation provided by a peer mentor may effectively bypass the harsh self-judgment that is often characteristic of these youths (Figure 2).

**Figure 3:**
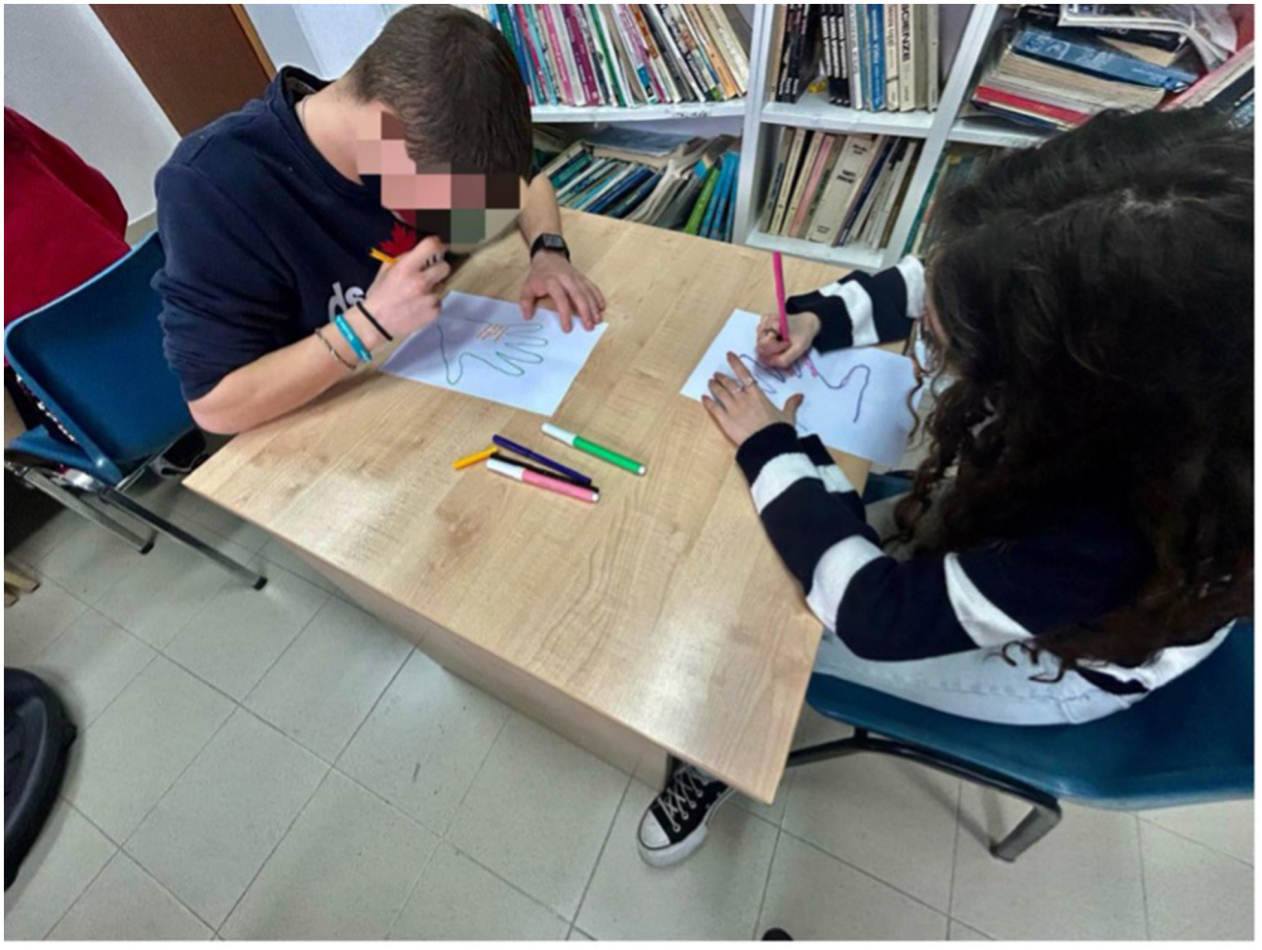
Putting yourself in someone else's shoes.

The final session aimed to consolidate group cohesion and was structured into two distinct, high-impact activities. The first activity was designed to promote interpersonal openness, enhance self-awareness, and facilitate the mutual exchange of thoughts, emotions, and bodily sensations through non-verbal contact. From an operational standpoint, participants were asked to hold hands with their eyes closed, focusing exclusively on their partner's heartbeat and the warmth of their hands. This sensory exercise made it possible to perceive whether the partner was in a state of tension or relaxation through somatic indicators such as sweaty hands or slight tremors. Following this interaction, participants traced the outline of their own hand on a blank sheet of paper. The sheets were then exchanged between partners, who wrote inside the drawn hand the specific physical and emotional sensations they had perceived during the experience. The results highlighted the achievement of a strong emotional and somatic attunement: participants were able to convey both positive and negative sensations to their partners, depending on their emotional state, thereby facilitating a deep and authentic interpersonal connection and significantly contributing to the development of relational and emotional responsibility. However, direct physical contact and the vulnerability inherent in the activity generated discomfort and difficulty for some participants. The initially high levels of physical embarrassment required careful and targeted emotional management by the mentors to prevent avoidance behaviors and ensure an adequate level of psychological safety. The request to close their eyes during physical contact triggered defensive reactions in several participants. Some refused, stating: “I don't want to close my eyes because I lose control, and that disturbs me.” This resistance highlighted a profound need for hypervigilance. However, those who engaged in the exercise reached a surprisingly deep level of emotional involvement. A particularly powerful interaction occurred between a mentor and a youth. During the debriefing, the mentor later reported: “I felt a sudden surge of repressed anger, as if something bad had happened to me personally. I realized I was absorbing his internal state.” The youth confirmed this perception by saying: “I can feel you're becoming agitated because my own anger is passing through our hands.” This exchange demonstrates how the exercise bypassed verbal barriers, allowing for a raw and authentic emotional connection (Figure 3).

**Figure 4:**
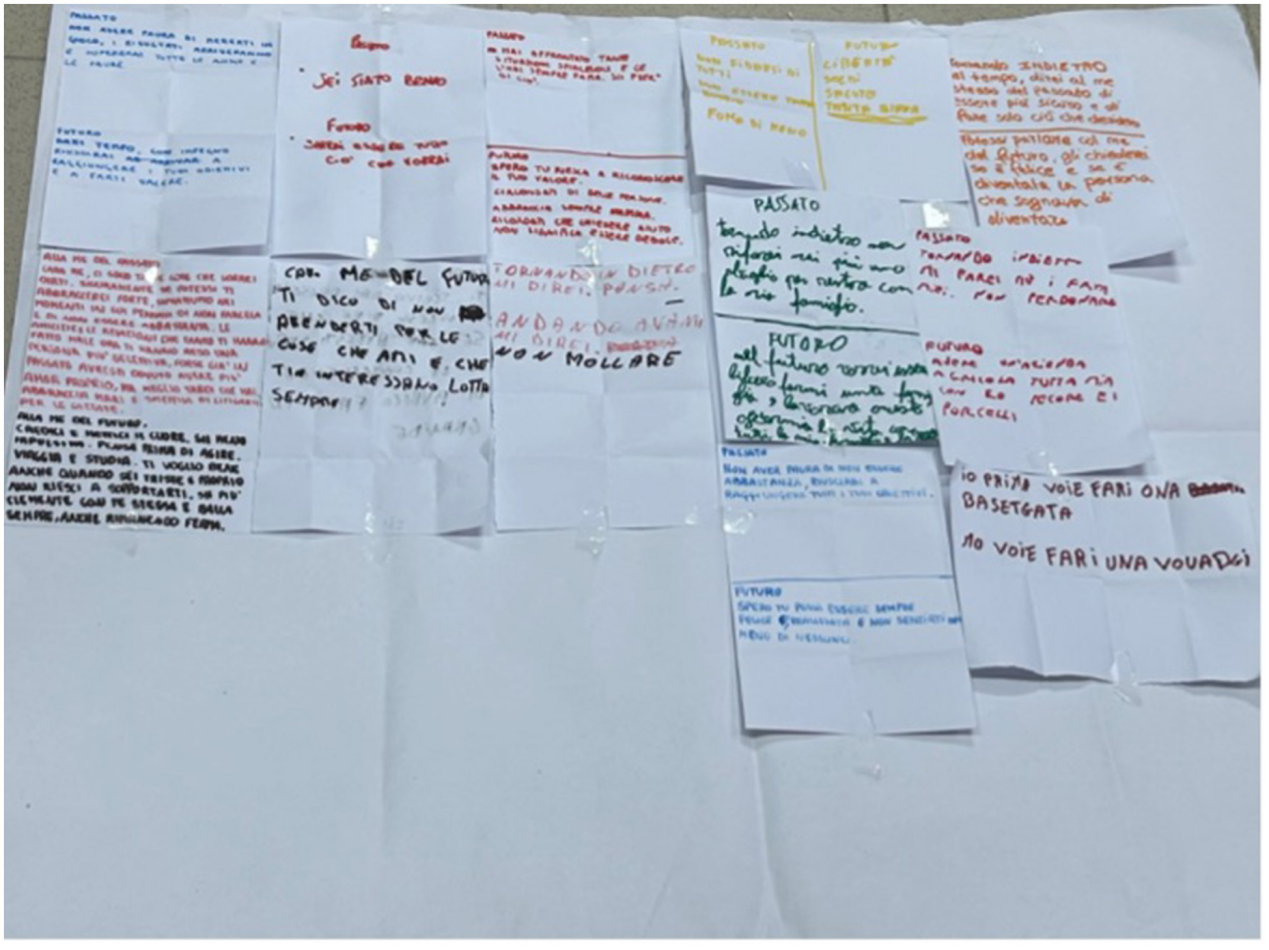
Offering a part of myself.

**Figure 5:**
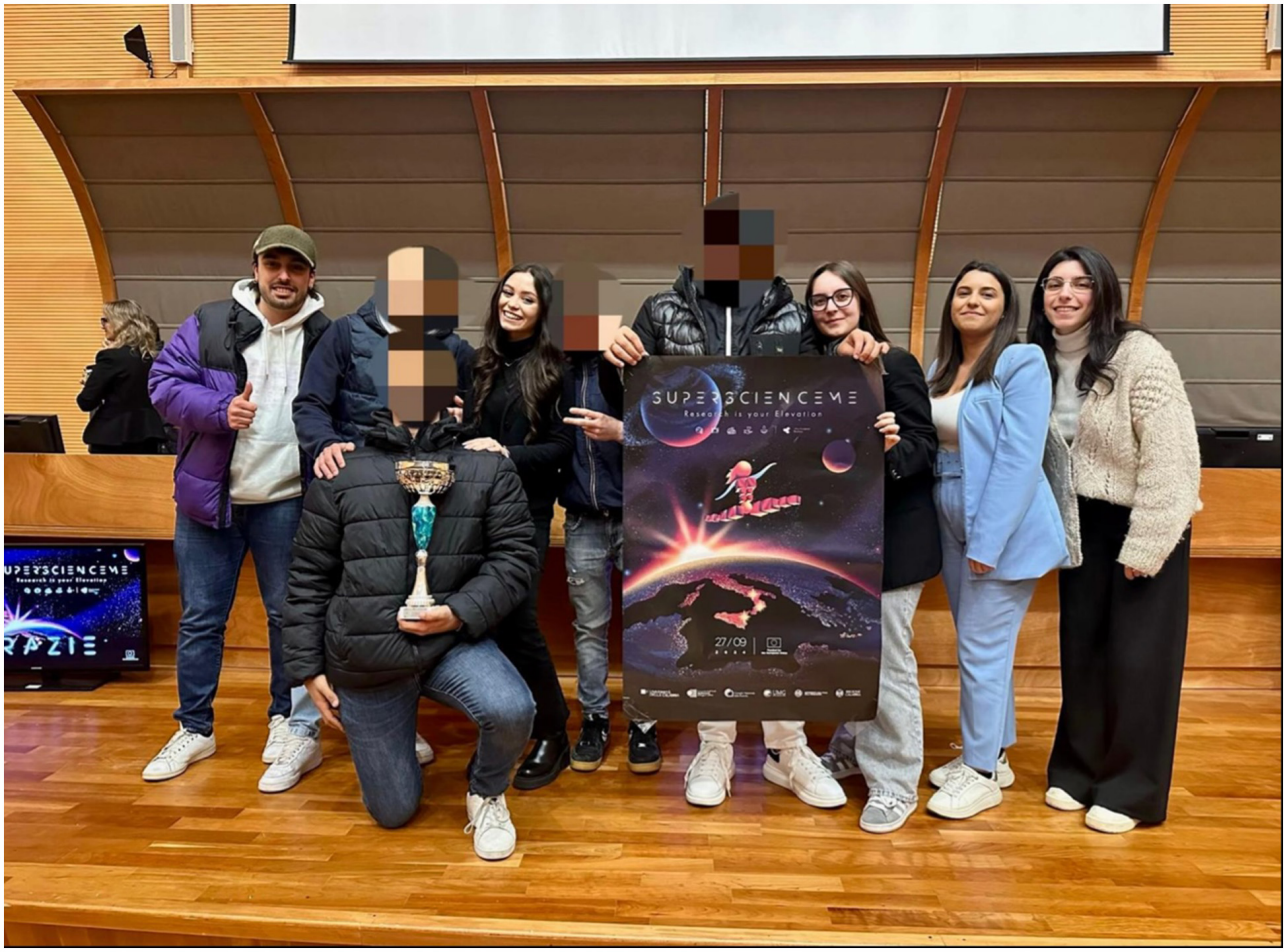
Our team during project award ceremony.

The final letter-writing activity represented the most comprehensive moment of integration of project's objectives, promoting awareness about the past and the future. Indeed, participants were invited to write a personal letter connecting past experiences with future goals. Subsequently, all the letters were placed on a single large poster and shared within the group. Participants read the collective texts and selected, by underlining them, the sentences or expressions from others that resonated most with them. Through the weaving together of these selected passages, the group co-constructed a single, shared letter capable of representing and connecting all participants. The final letter was then transformed into a recording composed of individual words chosen by each participant, resulting in a collective audio file. This final product was presented, together with the project report and the description of the activities carried out, during the competition. The collective sharing of personal narratives generated a strong sense of belonging and shared identity. At the same time, engaging with their past and with uncertainties about the future proved to be emotionally demanding. Some participants experienced significant difficulties in processing painful memories, highlighting the need for continuous and specialized emotional support to maintain a constructive and positive orientation toward the future. A key cross-cutting element throughout the entire process was the group work carried out at the end of each session, during which participants actively collaborated in constructing the final report to be presented during the competition. This co-construction process functioned not only as an operational tool but also as an important team-building experience, contributing to the strengthening of group cohesion and the sharing of common goals. A high level of instrumental collaboration was observed during the creation of the final poster. Participants spontaneously coordinated the sharing of tools, such as scissors and markers, demonstrating a fluid group synergy. There was also a visible effort to “claim” sentences from one another's letters, with the youths discussing which expressions were meaningful or powerful enough to be placed at the center of the collective work. The audio-recording phase was characterized by a complex emotional climate: long silences were punctuated by nervous laughter, revealing the embarrassment associated with being heard and recognized. As one mentor noted, “The atmosphere was a mix of pride and melancholy. While they were satisfied with the final product, a veil of sadness emerged as they realized that the journey together was coming to an end.” This bittersweet conclusion suggests that the project succeeded in fostering a meaningful relational bond that participants were reluctant to let go of (Figures 4, 5).

## 5 Discussion

Results highlighted key themes related to the experience and perceived impact of the intervention. Group activities, discussions, and guided reflections revealed increased juveniles' self-awareness, openness to others, future-oriented thinking, and accountability for antisocial behaviors. The intervention was experienced in a non-judgmental relational setting, facilitating the expression of emotions and personal experiences that are often difficult to share in institutional contexts.

In line with the ACEs model, cumulative exposure to trauma compromises emotional regulation processes, identity development, and interpersonal relationships, elements often associated with antisocial traits (Cook et al., 2005; van der Kolk, 2005; Hughes et al., 2017). From this perspective, the initial distrust and relational withdrawal observed among participants could be interpreted as adaptive responses to early adverse relational experiences, rather than as intentional expressions of oppositionality.

Another important result concerns the increased awareness of self and personal history. Through the narration of their past and future, participants reflected on their experiences and identity, recognizing the relationship between personal events, behavioral choices, and emotions. This process is particularly crucial in individuals with traumatic histories, as narrative reconstruction enables the integration of fragmented experiences and enhances personal resources and mentalization capacities (Fonagy and Target, 2001; Fonagy et al., 2002; Gorgellino et al., 2025; Jeong and Ha, 2026).

Furthermore, narrative sharing encouraged aspirations and ambitions related to the desire for change and improvement, the development of positive relationships, and social reintegration. At the same time, the construction of a coherent and future-oriented self-narrative represents a central mechanism in the process of moving away from deviant identities and behaviors (Maruna, 2001).

Overall, in line with the literature, the findings highlight the crucial role of significant relationships in adolescent development and behavioral change. In this sense, mentoring can be understood as a supportive emotional experience, providing a stable interpersonal context capable of modulating patterns of hyperactivation or deactivation of trauma-related dysregulation (Schore, 2001; van der Kolk, 2005).

In this frame, transforming feelings of guilt into responsibility is essential in restorative justice models, which emphasize understanding the consequences of crime and recognizing its impact on victims and the community (Zehr, 2002).

### 5.1 Limitations and future outcomes

Despite these encouraging preliminary findings, the project presents several limitations related to the exploratory, qualitative, and community-oriented nature of the intervention.

First, the study did not include standardized fidelity checks, structured implementation protocols, or formal statistical measurement and control procedures. As previously discussed, this methodological choice was consistent with the flexible, participatory, and relational aims of the intervention, but it limits the possibility of systematically assessing intervention adherence, reproducibility, and efficacy through quantitative indicators.

Additional limitations include the short duration of the intervention, the absence of follow-up measures capable of evaluating potential long-term effects on participants' behaviors and relational functioning, and the relatively small sample size, which restricts the generalizability of the findings. Consequently, the outcomes presented in this study should be interpreted as preliminary, descriptive, and practice-oriented observations rather than definitive evidence of effectiveness.

Nevertheless, the project represents a meaningful experience in the development of community-based rehabilitative practices involving vulnerable youth populations. Future implementations of the project could incorporate more structured methodological procedures, including standardized outcome measures, fidelity assessment protocols, longitudinal follow-up evaluations, and larger participant samples, in order to strengthen the scientific evaluation of the intervention and better assess its long-term impact and reproducibility across different contexts. If further implemented and developed beyond its current limitations, the project may contribute to fostering the following aspects: (a) Social Impact: The project represents an important field-training opportunity for future psychologists and criminologists, enabling them to develop practical, relational, and reflective skills through direct engagement with contexts of vulnerability and juvenile delinquency; (b) Economic Impact: The preventive and rehabilitative approach of the project may produce potential long-term economic benefits by reducing social and institutional costs associated with juvenile delinquency and recidivism; (c) Cultural Impact: The project fosters a culture of legality grounded in understanding harm, accountability, and the possibility of personal change, challenging stigmatizing views of young offenders and emphasizing the transformative role of educational and academic institutions within the community.

Despite the limitations, the *Vis à Vis* experience suggests the potential positive impact of meaningful relationships in promoting rehabilitative pathways among justice-involved juveniles.

The innovative component of the proposed project, both from an educational perspective for the university students involved and in terms of empowerment among the participating young people, made it possible to evaluate an intervention model that may be replicated in the future, while taking into account the identified limitations in order to further refine and strengthen the model within a continuous improvement framework.

## Data Availability

The original contributions presented in the study are included in the article/supplementary material, further inquiries can be directed to the corresponding author.
